# Identification of QTLs and a Candidate Gene for Reducing Pre-Harvest Sprouting in *Aegilops tauschii*–*Triticum aestivum* Chromosome Segment Substitution Lines

**DOI:** 10.3390/ijms22073729

**Published:** 2021-04-02

**Authors:** Jie He, Dale Zhang, Xian Chen, Yuge Li, Minjie Hu, Shaoguang Sun, Qing Su, Yarui Su, Suoping Li

**Affiliations:** School of Life Sciences, Henan University, Kaifeng 475001, China; 104752140073@vip.henu.edu.cn (J.H.); zhangdale@henu.edu.cn (D.Z.); 104753190830@vip.henu.edu.cn (X.C.); lygzixiu@henu.edu.cn (Y.L.); 104753190829@henu.edu.cn (M.H.); 104753201095@henu.edu.cn (S.S.); 104752160074@vip.henu.edu.cn (Q.S.)

**Keywords:** chromosome segment substitution lines (CSSL), Kompetitive allele-specific PCR (KASP), pre-harvest sprouting (PHS), quantitative trait loci (QTLs), RNA-Sequence, qRT-PCR

## Abstract

Wheat pre-harvest sprouting (PHS) causes serious losses in wheat yield. In this study, precise mapping was carried out in the chromosome segment substitution lines (CSSL) F_2_ population generated by a direct cross of Zhoumai 18 (PHS-sensitive) and *Aegilops tauschii* accession T093 (highly PHS-resistant). Three *Ae. tauschii*-derived quantitative trait loci (QTLs), *QDor.3D.1*, *QDor.3D.2*, and *QDor.3D.3*, were detected on chromosome 3DL using four simple sequence repeats (SSR) markers and 10 developed Kompetitive allele-specific PCR (KASP) markers. Alongside these QTL results, the RNA-Seq and qRT-PCR analysis revealed expression levels of *TraesCS3D01G466100* in the *QDor.3D.2* region that were significantly higher in CSSLs 495 than in Zhoumai 18 during the seed imbibition treatment. The cDNA sequencing results of *TraesCS3D01G466100* showed two single nucleotide polymorphisms (SNPs), resulting in two changed amino acid substitutions between Zhoumai 18 and line 495, and the 148 nt amino acid substitution of *TraesCS3D01G466100*, derived from *Ae. tauschii* T093, which may play an important role in the functioning of ubiquitin ligase enzymes 3 (E3) according to the homology protein analysis, which could lead to differential PHS-resistance phenotypes. Taken together, our results may foster a better understanding of the mechanism of PHS resistance and are potentially valuable for marker-assisted selection in practical wheat breeding efforts.

## 1. Introduction

Wheat pre-harvest sprouting (PHS) is the precocious germination of grain in a moist environment long before a crop’s harvest. PHS may cause a decrease in the end-use quality of grain due to starch and protein degradation, leading to severe wheat yield losses [1], and it often occurs before the grain-ripening season in humid and warm areas such as southwestern China [2]. Annual losses caused by PHS to wheat farmers are likely to exceed 1 billion USD worldwide [3]. To accommodate the growing demands of the international and domestic markets, it is imperative that wheat PHS resistance be strengthened to ensure and augment its yield and quality.

The phenomenon of PHS is influenced by a number of plant physiological factors. Dormancy is currently presumed to be the chief factor involved [4]. Seed dormancy, which can enable seeds to survive in adverse environments, such as in a wrong season or regeneration niche, is a complex quantitative trait. The lack of dormancy directly results in PHS under particular humidity and temperature conditions. Besides seed dormancy, grain color, and chemical coat composition, structure and ear morphology, as well as hormonal factors, could also affect PHS [5,6]. Although PHS is influenced by multiple genes and environmental conditions [7,8,9], annual variation in the latter could modulate the effects of alleles contributing to wheat’s tolerance of PHS. Hence, genotype-based selection could still promote this tolerance trait independently of the environment [10,11].

Most often, PHS is evaluated in both field and controlled environments using three methods: visual observation, alpha-amylase testing, and the Hagberg-Perten Falling Numbers (FN) assay [12,13,14]. For such research, misting chambers with intact wheat heads or a weighted germination index are commonly used to evaluate (susceptibility in the laboratory. However, because evaluating the phenotype is time- and labor-consuming, marker-assisted selection (MAS) is a better way to evaluate PHS because not only is it faster and requires less time, but it also is more accurate than phenotype evaluations.

A quantitative trait locus (QTL) is a locus (section of DNA) that correlates with variation in a quantitative trait in the phenotype of a population of organisms [15]. Therefore, finding reliable markers closely linked to quantitative trait loci (QTLs) that confer PHS resistance is crucial for marker-assisted selection [9,16]. To date, numerous QTLs for PHS resistance have been successfully mapped onto hexaploidy wheat chromosomes [17,18,19,20,21,22,23,24,25,26,27]. The first QTL found for grain dormancy in wheat was reported in 1993 [17]. Since then, many QTLs associated with PHS resistance were discovered on 21 chromosomes of the wheat genome [20,21,22,28,29,30], and major QTLs for PHS resistance were also detected on chromosomes 2B [22,25], 3A [31], and 4A [21,32,33,34]. Yet, only a few QTLs have been consistently detected across all wheat varieties because the wheat materials are from or grown in different environments [6,8,16]. Because the markers are often useful in the region where the marker was designed, the choice of widely applicable markers, able to detect individual polymorphisms in genes that directly impact PHS, is essential [11].

Until recently, a few genes associated with PHS in wheat had been identified across multiple studies, including *TaMFT/TaPHS1*, *TaMKK3*, *TaVp-1*, *Tamyb10*, *TaSdr*, *TaQSd*, and *TaDOG1* [1,35,36,37,38,39,40,41,42,43,44,45,46]. *TaMFT/TaPHS1* was identified as a potential gene for a PHS QTL-*QPhs.pseru-3A* by Nakamura et al., 2018 and later confirmed by *TaMFT* that was found in a 3AS QTL, for which three single nucleotide polymorphisms (SNPs) were found in the promoter and third intron region, located at the −222 and 646/666 positions, respectively. Notably, those lines carrying alleles at −222 and 646/666 were distinguished as having high resistance to PHS [45,47]. *TaMKK3* is another candidate gene in the *Phs1-4AL* QTL. It possibly encodes a mitogen-activated protein kinase kinase 3. The homologous gene *MKK* in *Arabidopsis* positively modifies abscisic acid (ABA) responsiveness [48]. So far, a resistance (*R*) gene has been identified in QTLs for both grain color and dormancy [28,34]. Recent studies suggest the *R* gene and *TaVp-1* encode *Tamyb10* transcription factors and embryo dormancy-related transcription factors, respectively [49]. In addition to those genes, others are considered candidate genes for PHS resistance because they are crucial participants in dormancy and/or germination in related or model plant species, including *TaSdr*, *TaQSd,* and *TaDOG1* [38,42,43,50].

However, the narrow genetic basis of wheat varieties has limited further improvements to wheat yield and quality, while the wild relatives of wheat offer valuable genetic resources for wheat germplasm improvement. The introduction of this germplasm is a key way to broaden the genetic basis for variation in wheat grain yield and resolve its narrow wheat heredity. *Ae. tauschii* Cosson (DD, 2n = 2x = 14) is considered the progenitor of common wheat; because it contains abundant beneficial genes to resist PHS, disease, and stress, it is recognized as a potential gene donor for breeding wheat [51]. In the early stage of our study, chromosomal segment substitution lines (CSSLs) were obtained via direct hybridization between the Zhoumai 18 and *Ae. tauschii* T093 [52]. Fortunately, *QDor-3D*, an *Ae. tauschii*-derived stable QTL for PHS, was detected in the CSSLs and found located within the marker interval of *Xgpw5094*–*Xcfd223*, for which the value of phenotypic variation explained (PVE) was 11.77% [52]. From the resulting CSSL BC_3_F_4_, one of the individuals, 144148-1, had high resistance to PHS and was introgressed with the stable QTL, and so it could be used as key plant material for the precise mapping of *Ae. tauschii* genes controlling PHS in wheat. In the present study, 144148-1 was further hybridized with Zhoumai 18 to obtain the F_2_ segregation population, whose PHS resistance trait displayed significant phenotypic variance. However, the genetic and molecular mechanisms of differential PHS resistance in this F_2_ population were unclear. So, our objectives were threefold: to carry out the precise mapping of PHS resistance genes in the mapped F_2_ population, to develop user-friendly DNA markers, and to find candidate genes related to PHS for MAS.

## 2. Results

### 2.1. Phenotypic Characterization of PHS

**Figure 1:**
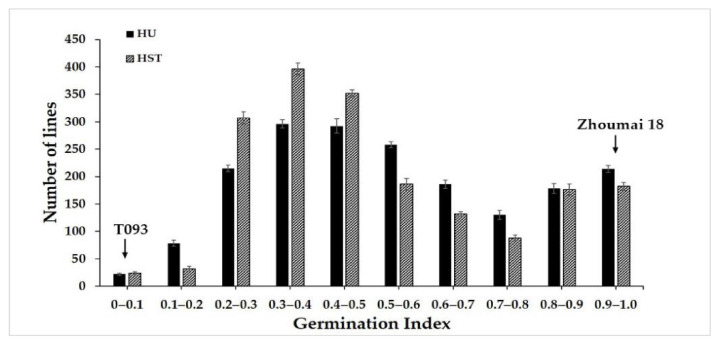
Frequency distributions of the germination index values of the F_2_ population of wheat tested at two field sites. HU denotes Henan University; HST denotes the Henan Agriculture High-Technology Science and Technology Park.

For the advanced backcross 1884 F_2_ population, as well as the T093 and Zhoumai 18 wheat, their respective germination index values were calculated in 2016 to evaluate their PHS resistance in two experimental fields. The statistical analysis showed that T093 had significantly greater resistance to PHS than did Zhoumai 18. The results of ANOVA showed that the germination index (GI) did not differ significantly between environments (F = 0.398, *p* > 0.05). The trend in resistance to seed germination of the lines in the two experimental fields was basically the same. The germination index (GI) in the F_2_ population showed continuous variation (Figure 1), which suggested PHS was a typical quantitative trait. Further, the PHS resistance of the F_2_ population displayed significant phenotypic variance. The GI values of the population ranged from 0 to 1, its average being 0.54 (Figure 1).

### 2.2. Precise Mapping of Qphs-3D Using the SSR and KASP Markers

**Figure 2:**
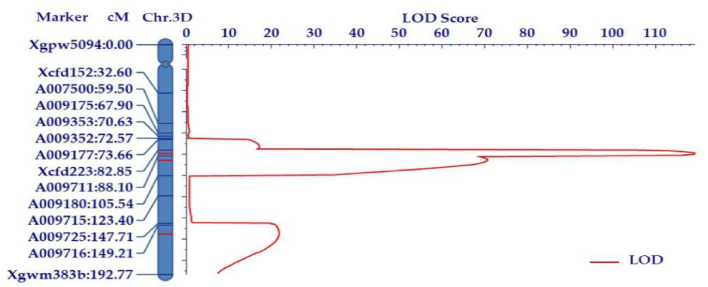
Linkage groups assigned to the chromosome arms 3DL in the chromosomal segment substitution lines (CSSL) of Zhoumai 18 and T093. DNA markers and their positions are shown along the chromosome arms. The distance between adjacent markers is given in the cM units of Kosambi. The threshold logarithm of the odds (LOD) is 2.61.

Our previous mapping revealed a major PHS QTL (*Qdor.3D)* within the marker interval of *Xgpw5094*–*Xcfd223*. In the present study, four polymorphic simple sequence repeats (SSR) primers were screened within this interval. Based on the bulk segregation analysis (BSA) and the wheat 660K array results, 10 SNPs were selected between Zhoumai 18 and T093 in the targeted QTL region to convert Kompetitive allele-specific PCR (KASP) markers, and their polymorphism between parents was confirmed using the BSA combined with the 660k SNP array. These KASP markers can be useful for identifying the alleles of T093 and Zhoumai 18. Subsequently, the 10 polymorphic KASP markers and four SSR markers were genotyped onto the F_2_ population and mapped in 3DL. The main peak of the logarithm of the odds (LOD) occurred in the region of 86 cM, situated between *Xcfd223* and *Xgwm383b* (Figure 2). Three QTLs were identified in this interval, explaining 4.74–24.16% of the phenotypic variation in the F_2_ population (Table 1). The LODs of the three QTLs were 119.44, 70.28, and 22.17, respectively, and their corresponding additive effects were −16.61, −12.59, and −6.02. This indicated that all additive QTLs were derived from *Ae. tauschii* T093, which suggested these *Ae. tauschii*-derived loci had positive effects on PHS resistance. At the same time, a 157 cM linkage map on a 3D chromosome was constructed. The main QTL, named *QDor-3D.1,* having the highest LOD value (119.14), was mapped to an interval flanked by the SSR marker *Xcfd223* and the KASP marker *A009711*. The physical distance between *Xcfd223* and *A009711* was 2.1 Mb, while the genetic distance between them was approximately 5.25 cM. *QDor-3D.1* exerted a significant effect in that it explained 24.16% of the variance in the resistance phenotype (Table 1). *QDor-3D.2*, which was closely linked with *QDor-3D.1*, was mapped to an interval flanked by *A009711* and *A009180*. The physical distance of *QDor-3D.2* between KASP marker *A009711* and KASP marker *A009180* was 6.9 Mb, and this explained 15.49% of resistance phenotypic variance. Lastly, *QDor-3D.3* was mapped to an interval flanked by the KASP marker *A009716* and the SSR marker *Xgwm383b*. Of the three QTLs, *QDor-3D.3* explained the least variance (4.74%) in the resistance phenotype.

### 2.3. RNA-Seq and qRT-PCR Assay

**Figure 3:**
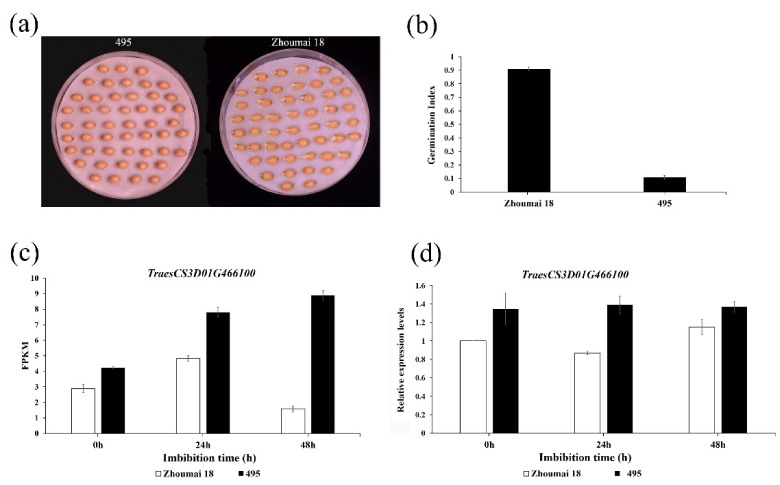
Expression of *TraesCS3D01G466100* promotes seed dormancy in wheat. (**a**) Germinated seeds of Zhoumai 18 and line 495. Photographs were taken at 48 h after imbibition. (**b**) Gemination index of Zhoumai 18 and line 495. (**c**) Fragments per kilobase of exon model per million mapped fragments (FPKM) of *TraesCS3D01G466100* in the embryos of Zhoumai 18 and line 495. (**d**) Relative expression levels of *TraesCS3D01G466100* in the embryos of Zhoumai 18 and line 495. Error bars show the standard deviation of three replications.

RNA-Seq was used to identify differentially expressed genes (DEGs) after wheat seeds’ imbibition. A total of 25.22 Gb of clean data was obtained for the 18 samples after filtering the low-quality reads. The percentage of bases with Q30 was at least 93.43%. The DEGs were detected using the R procedure in Bioconductor package EBseq_DESeq. The gene with |log2(FC)| ≥ 2 (FDR = 0.01) was considered to be differentially expressed. In comparing the genes at 0, 24, and 48 h of Zhoumai 18 and the CSSLs line 495 after germination, a total of 12 DEGs were identified and located in the PHS QTL interval on chromosome 3D: *TraesCS3D01G461400*, *TraesCS3D01G462600*, *TraesCS3D01G464200*, *TraesCS3D01G466100*, *TraesCS3D01G467100*, *TraesCS3D01G467200*, *TraesCS3D01G467300*, *TraesCS3D01G467500*, *TraesCS3D01G467600*, *TraesCS3D01G468500*, *TraesCS3D01G470900*, and *TraesCS3D01G473200*. The results showed that Zhoumai 18 is sensitive to PHS, whereas line 495 is resistant to PHS, whose respective germination index was 0.91 and 0.11 (Figure 3a,b). At the same time, according to the Swiss-protein and gene ontology (GO) annotations, *TraesCS3D01G466100* functioned as an E3 ubiquitin-protein ligase in rice and participated in the seed dormancy process, making it a potential candidate gene. Dry seeds and imbibed seeds of Zhoumai 18 and line 495 were evaluated after 24 and 48 h, and their transcriptome and fluorescence quantitative PCR results are shown in Figure 3c,d. Both the fragments per kilobase of exon model per million mapped fragments (FPKM) and relative expression level of *TraesCS3D01G466100* were always lower in Zhoumai 18 than in line 495, in all three stages. Therefore, its greater expression may promote dormancy and/or inhibit germination. In the target region, the gene *TraesCS3D01G466100* was located at 569,781,286–569790580 bp, encoding a protein functionally similar to RING finger E3 ubiquitin ligase, whose function is the monoubiquitinating of histone H2B. Histone H2B monoubiquitylation plays an important role in seed dormancy regulation levels. Members of this RING finger E3 ubiquitin ligase family were identified as involved in the chromatin remodeling of the seed dormancy mechanism in *Arabidopsis* [53].

### 2.4. TraesCS3D01G466100 cDNA Sequencing and Protein BLAST

**Figure 4:**
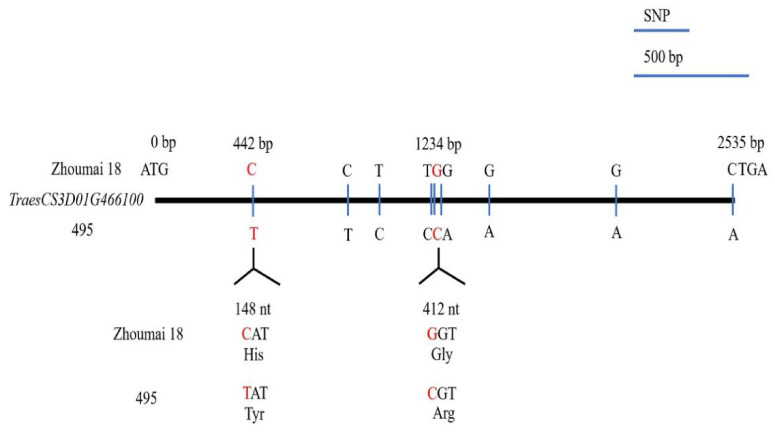
Sequence variation in the *TraesCS3D01G466100* cDNA structure of Zhoumai 18 and line 495 wheat for resistance to pre-harvest sprouting (PHS). The single nucleotide polymorphisms (SNPs) in the coding DNA sequence (CDS) are indicated by vertical lines; each SNP is indicated by the enlarged codons and their encoded amino acids.

**Figure 5:**
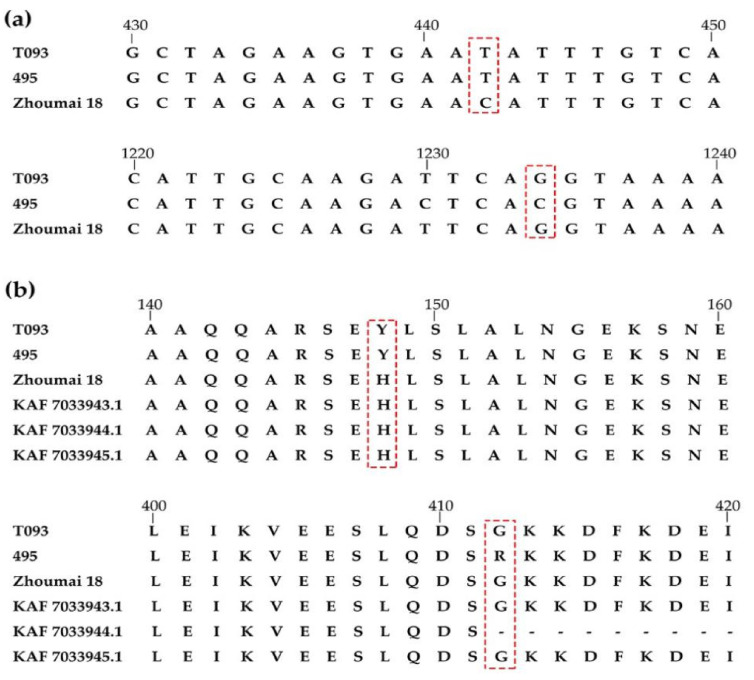
Analysis of *TraesCS3D01G466100* cDNA and alignment of protein sequences. (**a**) The *TraesCS3D01G466100* cDNA structure and variations among the alleles from T093, line 495, and Zhoumai 18. The bases in virtual frames result in the change of amino acids in predicting protein sequence in T093, line 495, and Zhoumai 18. (**b**) Alignment of protein sequences among homologous TraesCS3D01G466100 from T093, line 495, and Zhoumai 18, and three other *Triticum aestivum* (KAF7033943.1, KAF7033944.1, and KAF7033945.1). The amino acids in virtual frames indicate the variation among line 495 and T093, Zhoumai 18, and three other *Triticum aestivum*.

The full-length cDNA of *TraesCS3D01G466100* amplified from seedlings of Zhoumai 18, T093, and line 495 were sequenced, from which a 2535-bp full-length cDNA was obtained by Geneious 4.8.5. This cDNA sequence encoded a functional peptide of 844 amino acids. In comparing the cDNA sequences of *TraesCS3D01G466100* between Zhoumai 18 and line 495, nine SNPs were identified (Figure 4). Just two different amino acids, located at 148 nt (442 bp) and 412 nt (1234 bp), were identified in the predicted transcript (Figure 4 and Figure 5a). The 148 nt of the full-length cDNA sequence corresponded to His(H) in Zhoumai 18 but Tyr (Y) in line 495 and T093, while 412 nt was Gly (G) in Zhoumai 18 and T093 and Arg(R) in line 495. These discrepancies are nonetheless consistent with our results for the DNA sequence alignment. The corresponding cDNA sequence had C in Zhoumai 18 but T in line 495 at 442 bp, and likewise, G in Zhoumai 18 but C in line 495 at 1234 bp.

## 3. Discussion

Using PHS-resistance genes is crucial for wheat breeding practices. The development of PHS resistance lines offers one basis for the enrichment of genetic resources in wheat breeding programs; accordingly, introducing genes from wheat-related species could provide an effective way to significantly improve wheat grain quality and its resistance to PHS. Liu et al. and Yu et al. both found that genes from *T. turgidum* and *Ae. tauschii* can be applied to enhance the PHS resistance of wheat [54,55]. A direct cross of *Ae. tauschii* with common wheat was reported in two studies [56,57]. Moreover, the CSSL population has been widely used for the fine mapping of complex traits in wheat, rice, maize, and other crops since 1987 [52,58,59,60]. In our previous study, we developed a population of CSSLs by relying on synthetic octaploid wheat as a bridge to identify a *QDor-3D* for PHS [52]. The direct hybridization of wheat and *Ae. tauschii* can effectively avoid interference from the A and B genomes, and the excellent genetic background of wheat makes the CSSLs more convenient for wheat breeding. Furthermore, in this study, an advanced F_2_ population was obtained from the individual 144148-1 of CSSLs backcrossed with Zhoumai 18 and then self-crossed. Therefore, only D genome introgression existed in the lines, and the chromosome would be further recombined via linkage and interchange processes. Moreover, any interference from genetic background variation was eliminated, which meant the reserved lines still retained better agronomic traits, and the QTL was guaranteed to be more accurate.

As it stands, most studies have indicated that when multiple genes are controlling the same trait, they are often closely linked, and so the QTL close linkage is a common phenomenon. For example, Fofana et al., 2009 identified three QTLs: *QCL.crc-3A*, *QCL.crc-3B*, and *QCL.crc-3D* associated with the red coloring of the seed coat. Work by Monna et al., 2002 produced high-resolution linkage mapping of *Hd3,* finding that *Hd3a* and *Hd3b* were tightly linked in that region. Fortunately, by expanding the population to generate a secondary segregation population, QTL mapping can more accurately decompose those QTL clusters under genetic background control. For example, Kumar et al. also identified three QTLs using GR and PG measures, namely *QPhs.spa-3A*, *QPhs.spa-3B*, and *QPhs.spa-3D*, which were localized to the same region as in the study of Fofana et al., mentioned above [61,62]. In our study, *QDor-3D.1*, *QDor-3D.2*, and *QDor-3D.3* were all identified using a large segregating population derived from advanced backcross progeny. *QDor-3D.1* and *QDor-3D.2* were tightly linked in the *QDor-3D* region, which was positioned between *Xcfd223* and *A009180*. More importantly, *Xcfd223* was detected in both studies by Fofana et al. and Kumar et al. above, which suggests the QTL interval on 3DL figures prominently in the reduction of wheat germination. The co-located QTLs conferring PHS resistance could be found at analogous positions on 3D by respectively using CSSLs populations and the advanced F_2_ segregating population, thus providing strong evidence of the existence of nearby candidate genes for the PHS-resistance trait. Compared with previous studies, the PHS QTL region was saturated with more markers in our study, and so, it would be more convenient to further study the mechanism underpinning PHS resistance with these QTLs.

Several genes have been verified as associated with PHS in wheat in recent reports, including *TaMFT/TaPHS1*, *TaMKKK3*, *Tamyb10*, and *TaVp1* in wheat and *TaSdr*, *TaQSd*, and *TaDOG1* in related crops (i.e., barley, maize, rice) or model (*Arabidopsis*) organisms [1,35,36,37,38,39,40,41,42,43,44,45,46]. In recent years, RNA-Seq has emerged as a powerful technique for DEG identification at the transcriptome level, which could provide insights into the molecular mechanism(s) underpinning PHS resistance [63]. In addition, the quantitative analysis of DEGs can be further verified by real-time quantitative PCR. According to the DEGs in the *QDor-3D.2* region detected by RNA-Seq, the expression level of *TraesCS3D01G466100* differed significantly between Zhoumai 18 and line 495, and *TraesCS3D01G466100* might be a RING finger-dependent E3 ubiquitin-protein ligase in rice that also participates in seed dormancy process according to its Swiss Prot and GO annotations. E3 ubiquitin-protein ligase homologous alignment with three other *T. aestivum* encoded on 3D chromosome indicated the locus 148 nt in line 495 was the same with his parent *Ae. tauschii* T093 yet different from Zhoumai 18 and three other *T. aestivum* (KAF 7033943.1, KAF 7033944.1, and KAF 7033945.1). The change in amino acid results from the sense mutation of the 442 positions. Here, the base was T at 442 bp in line 495, which was the same as in T093, which indicated that base T was most likely from T093 during introgression. The changed base causes the change in the amino acid, which then affects the function of E3, resulting in the significant QTLs related to PHS and the difference in the GI between Zhoumai 18 and line 495. Interestingly, in line 495, another base C at 1234 bp differs from both Zhoumai 18 and T093, which may be a mutation during introgression.

Ubiquitin-mediated protein degradation is an essential protein post-translational modification in eukaryotic cells, one related to the fine-scaled regulation of their biological growth and environmental adaptation by organisms. E3 (ubiquitin-ligating enzyme) along with E1 (ubiquitin-activating enzyme) and E2 (ubiquitin-conjugation enzyme) are required for target protein ubiquitination, which eventually leads to the degradation or relocation of that targeted protein. In recent years, mounting studies have shown that E3 plays a positive or negative regulatory role in a variety of disease resistance signaling pathways, mainly in the ABA signal transduction pathway. Surprisingly, however, the effect of E3 on PHS resistance is rarely reported on, especially in wheat. Liu et al.,2007 found that *HISTONE MONOUBIQUITINATION1* (*HUB1*) encodes a C3HC4 RING finger protein. Because *HUB1* probably regulates the monoubiquitinating of histone H2B that influenced PHS-related gene expression in *Arabidopsis*, it has been proposed that chromatin remodeling could be critical for the regulation of seed dormancy [53]. Zheng et al., 2018 also found that a RING-type ubiquitin E3-ligase RHA2b targets MYB30 for degradation to modulate ABA signaling in *Arabidopsis* [64]. Another study showed that *OsHUB1/2* in rice was homologous to *Arabidopsis HUB1/2* and that treatment with hormones (JA, SA, ET, or ABA) induced the transcription of *OsHUB1/2*. This way, *OsHUB1/2* may interact with SPIN6 to regulate the SPL11-mediated rice immunity and cell death [65]. A database search revealed the sequence homology of *HUB1* and *HUB2* is based on a conserved gene that is required for H2B monoubiquitination to occur in different kinds of organisms, including *Medicago truncatula* (*Mt_Bre1*), rice (*O. sativa*; *Os_Bre1A* and *Os_Bre1B*), yeast (*Sc_Bre1*), and even humans (*Hu_Bre1A* and *Hu_Bre1B*) [66,67,68,69]. Nevertheless, this type of gene has never been found associated with PHS resistance in wheat until now (Figure 5b). We therefore postulate *TraesCS3D01G466100* is a novel PHS resistance-related gene critical for the establishment of seed dormancy in wheat. This provides a new way forward for elucidating the mechanism of resistance to PHS in wheat. We anticipate it will be a valuable potential gene for breeding PHS-resistant wheat crops.

## 4. Materials and Methods

### 4.1. Plant Materials and Mapping Populations

**Figure 6:**
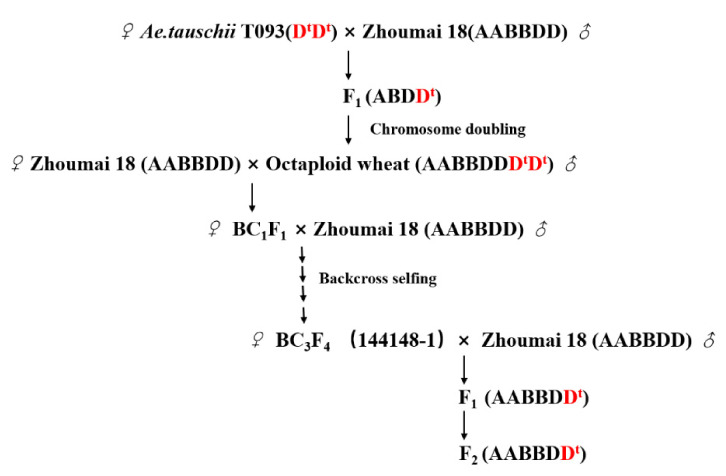
Crossing scheme used to obtain the F_2_ population of wheat in this study. D^t^ denotes the genome of *Aegilops tauschii*.

In our previous study, the CSSLs were developed by direct hybridization between *Ae. tauschii* accession T093 that featured strong resistance to PHS and the recurrent parent Zhoumai 18, whose susceptibility to PHS is high [52,70,71]. Here, individual 144148-1 with stable PHS resistance in the CSSLs was backcrossed again with Zhoumai 18 and then self-crossed. In this way, a total of 1884 F_2_ plants were finally generated for further investigation in this study (Figure 6).

### 4.2. Assessment of PHS in the Wheat F_2_ Population

To assess their PHS resistance, the F_2_ population, Zhoumai 18, and T093, were grown in 2015 in two places, Henan University (HU) and Henan Agriculture Hi-Tech Science and Technology Park (HST), respectively, both in China. The PHS resistance of the F_2_ population was evaluated in 2016. In the field experiment, all the lines were planted in 1.2-m-long single-row plots with 10 cm between plants and a 30-cm row gap. Twelve seeds were sown in each row. Five spikes were removed from each wheat plant after it attained physiological maturity. After all experimental plants were harvested, their spikes were dried for seven days at room temperature and stored in a refrigerator at −25 °C to maintain their dormancy until the germination test was conducted, following the methodology of Liu et al. [31]. Fifty seeds from each spikelet were sterilized with 5% sodium hypochlorite and then rinsed five times with sterile water until clean. These seeds were then placed in Petri plates, on moistened filter paper with sterilized water, at 22 °C in the dark. Over the next seven days, the germinating seeds were counted, and any germinated seeds found with radicle protrusions were immediately discarded. The phenotyping was conducted as described by James et al. [17]. The germination tests consisted of three replicates, each consisting of seeds from a different spike. The germination index (GI) was calculated as follows:

GI = (7 × n_1_ + 6 × n_2_ +……. + 1 × n_7_)/(total seeds × 7) (1)

where n_1_ is the number of germinated seeds during the first day, n_2_ is the number of germinated seeds during the second day, and total seeds are the number of total observed seeds in each Petri plate. The one-way analysis of variance (ANOVA) of GI from two environments was conducted on the mean of the three replications using SPSS Statistics 19.0. The mean GI values of the two sites were used for the next QTL analysis.

### 4.3. DNA Extraction and Marker Analysis

The DNA of fresh, healthy leaves was isolated by the modified cetyltrimethylammonium bromide (CTAB) method [72] and dissolved in sterile distilled water. Both DNA purity and quality were measured with a nanophotometer (Implen Inc., Los Angeles, CA, USA).

Because our previous study found that a major PHS QTL (*Qdor.3D*) was located within the marker interval of *Xgpw5094*–*Xcfd223* [52], this study further screened polymorphic SSR markers within this interval. Four polymorphic SSR primers, *Xgpw5094*, *Xcfd223*, *Xcfd152*, and *Xgwm383b*, were synthesized and designed based upon the genetic map of the D genome, in GrainGene 2.0 software (http://wheat.pw.usda.gov (accessed on 20 March 2021)), and the *Ae. tauschii* genome sequences [73,74] (Table 2). The PCR amplification for these SSR markers was conducted according to Röder and Bendich [75]. Specifically, the PCR cycles began with an initial denaturation at 95 °C for 3 min, then 35 cycles of denaturation at 95 °C for 30 s, annealing at 58 °C for 45 s, and extension at 72 °C for 1 min, followed by a final extension at 72 °C for 10 min. The ensuing PCR products were separated from 8% non-denaturing polyacrylamide gel sand and visualized with silver staining [76].

### 4.4. Bulk Segregation Analysis (BSA) with the 660K Chip Array and KASP Marker Development

The pooled genomic DNA from 30 F_2_ lines with high PHS resistance and likewise from 30 F_2_ lines with high PHS sensitivity, along with the DNA from T093 and Zhoumai 18, were used for the wheat 660K SNP chip analysis carried out by Beijing Golden Marker Co., Ltd. (Beijing, China). The data quality control was performed by Axiom Analysis Suite to provide a clean sample of SNP typing data. The calling and clustering of SNP genotypes were performed in Genome Studio Polyploid Clustering v 1.0 software. The correspondence of polymorphic SNPs in the BSA to chromosomes was based on the 660K physical map. The polymorphic SNPs between the high and low PHS-resistant bulks associated with the target QTLs on chromosome 3D in the marker interval *Xgpw5094*–*Xcfd223* were successfully converted into 10 KASP markers for the fine mapping [77] (Table 3).

### 4.5. QTL Analysis

For the QTL analysis, QTL IciMapping v4.0 was used with its composite interval mapping function [78]. Four SSR markers and 10 KASP markers were assessed using the map function, with the logarithm of the odds (LOD) threshold set to 2.61. QTLs were detected if their LOD score was above 2.61. The nnTwoOpt algorithm was used for the markers in a given group, and then the rippling step was applied to obtain a linkage map of the F_2_ population. Finally, the PHS QTL regions were identified by running the BIP function using the inclusive composite interval mapping with an additive effect (ICIM-ADD). This QTL analysis was conducted for the PHS phenotypes of all 1884 members of the F_2_ population.

### 4.6. RNA Isolation and Sequencing

For RNA-Seq, dried seeds and treated seeds were sterilized. Seeds of Zhoumai 18 and line 495 were surface-sterilized as described above for the germination index, and these were imbibed between filter papers in Petri plates at room temperature under darkness. Dry seeds and imbibed seeds after 24 and 48 h were frozen in liquid nitrogen, then stored at −80 °C until their RNA extraction.

Total RNA was isolated with the Spin Column Plant Total RNA Purification Kit by following the manufacturer’s protocol (Sangon Biotech, Shanghai, China). RNA degradation was checked on 1% agarose, and contamination was checked by a nanophotometer spectrophotometer (Implen, Los Angeles, CA, USA). The quantification of RNA was performed using the Qubit RNA Assay Kit with a Qubit 2.0 fluorometer (Life Technologies, Carlsbad, CA, USA). Next, RNA integrity was checked using the RNA Nano 6000 Assay Kit of the Agilent Bioanalyzer 2100 system (Agilent Technologies, Santa Clara, CA, USA).

Sequencing libraries were generated for sequencing on the Illumina Novaseq 6000 platform (Illumina Inc., San Diego, CA, USA), by Biomarker Technology, in Beijing. The mRNA was purified from total RNA by poly-T oligo-attached magnetic beads. The cDNA was then synthesized by short mRNA fragments with PCR amplification used to enrich the cDNA templates. Finally, the libraries were sequenced. Three biological replicates were used for each of the sampling points.

### 4.7. Gene Expression Using Quantitative Real-Time PCR

According to the QTL mapping analysis, the gene related to PHS resistance was located in 564.6–573.6 Mb on chromosome 3D based on the IWGSC_RefSeq_v1.0 wheat genome. Combined with the transcriptome results, 12 DEGs were screened in the same interval. The high expression of *TraesCS3D01G466100* was positively correlated with PHS resistance, and it was selected for the next experiment.

Real-time PCR was carried out to determine the expression levels of genes. The qRT-PCR was conducted on an ABI LightCyler QuantStudio 6 instrument, using the HiScript Reverse Transcriptase (RNase H) and 5 × HiScript Buffer (Vazyme, Nanjing, China). Each reaction was performed in a 20 µL mixture containing 10 µL of SYBR Green Master Mix; three biological replications were performed. The actin gene served as the internal control, and the specific primers were designed in Primer Premier 5.0 (Table 4). Data are presented here as relative transcript levels and calculated using the 2^−ΔΔCt^ method [79].

### 4.8. TraesCS3D01G466100 cDNA Sequencing and Protein BLAST

Total RNA was reversed to cDNA by a RevertAid First Stand cDNA Synthesis Kit (Fermentas). The primers for *TraesCS3D01G466100* cDNA on the 3D chromosome were designed based on *Ae. tauschii* by a National Center for Biotechonology Information (NCBI) homologous BLAST, using Primer Premier 5.0 (Table 3). The cDNAs of Zhoumai 18, line 495, and T093 were used as templates to perform the PCR amplification. Next, the genes were cloned into a *pEASY*-Blunt simple vector and transformed into Trans-T1 *Escherichia coli* cells (TransGen, Beijing, China). Three positive clones were sequenced to ensure the accuracy of nucleotide sequences (Sunya, Zhengzhou, China). For their homology alignment, the multiple sequences were compared and analyzed using Geneious v4.8.5. These E_3_ ubiquitin-protein ligases were all derived from a 3D chromosome of *T. aestivum.*

## 5. Conclusions

An advanced F_2_ population was spawned from the individual 144148-1 of *Ae. tauschii* (T093)—*T. aestivum* (Zhoumai 18) CSSLs backcrossed with Zhoumai 18 and then self-crossed. *QDor.3D.1*, *QDor.3D.2,* and *QDor.3D.3* were identified on the long arm of chromosome 3DL, using an advanced backcross F_2_ mapping population, for which *QDor.3D.1* and *QDor.3D.2* were tightly linked. Their additive effects revealed that these additive QTLs were derived from *Ae. tauschii* T093, which means these *Ae. tauschii*-derived loci positively influence PHS resistance. RNA-Seq and RT-PCR results indicated that *TraesCS3D01G466100* was probably the candidate PHS-related gene. The functional annotation of *TraesCS3D01G466100* pointed to an E_3_ ubiquitin-protein ligase, encoding a C3HC4 RING finger protein. The cDNA and predicted protein sequence analysis showed two amino acids altered between Zhoumai 18, line 495, and T093. Moreover, homology protein BLAST suggested the synonymous mutation site of 148 nt amino acids in line 495 was derived from the hybridization of *Ae. tauschii*. Because the RING finger-dependent E_3_ ubiquitin-protein ligase has not yet been found related to PHS resistance in wheat, we propose that the *TraesCS3D01G466100* studied here is a novel PHS resistance-related gene in wheat. Both the QTLs and this newly identified *TraesCS3D01G466100* should enrich the genetic resources available for wheat breeding aimed at mitigating PHS and could provide an important, fresh basis for agricultural practices promoting PHS resistance in cereal crops.

## Figures and Tables

**Table 1:** Quantitative trait loci (QTL) peak location, left and right markers, LOD values, phenotypic variation explained (PVE), and additive effect of QTL on chromosome 3DL estimated in the F_2_ population in 2016.

QTL	Chromosome Arm	QTL Peak Location (cM ^a^)	Left Marker	Right Marker	LOD ^b^	PVE ^c^ (%)	Additive Effect
*QDor-3D.1*	3DL	86.00	*Xcfd223*	*A009711*	119.44	24.16	−16.61
*QDor-3D.2*	3DL	92.00	*A009711*	*A009180*	70.28	15.49	−12.03
*QDor-3D.3*	3DL	157.00	*A009716*	*Xgwm383b*	22.17	4.74	−6.02

^a^ cM, centimorgans; ^b^ LOD, logarithm of odds value; ^c^ PVE (%), the percentage phenotypic variance explained by the QTL.

**Table 2:** Primer sequences of the simple sequence repeats (SSR) assays developed in this study.

Marker’s Name	Chromosomes	Genetic Position (cM)	Physical Position (Mb)	Primer Sequence (5′–3′)
*Xgpw5094*	3D	0	none	GACGATCAACAGCGAGTCAA
				TTACAATCTCACCCTGGCAA
*Xcfd152*	3D	32.60	538177121	TGGAAGTCTGGAACCACTCC
				GCAACCAGACCACACTCTCA
*Xcfd223*	3D	82.85	564608827	AAGAGCTACAATGACCAGCAGA
				GCAGTGTATGTCAGGAGAAGCA
*Xgwm383b*	3D	192.77	none	ACGCCAGTTGATCCGTAAAC
				GACATCAATAACCGTGGATGG

**Table 3:** Primer sequences of the Kompetitive allele-specific PCR (KASP) assays developed in this study.

Marker’s Name	Genetic Position (cM)	SNP Probe	Physical Position (Mb)	T093 Allele	Zhoumai 18 Allele	Allele-Specific Primer (5′–3′)
*A007500*	59.5	*AX108732216*	541371314	C/C	G/G	FAM: TTGATAGGTTCGCATAAATATATCACAC VIC: TTGATAGGTTCGCATAAATATATCACAG Com: ATGACTCAAGGCAGAAGGGTGCAAA
*A009175*	67.9	*AX110029380*	553359206	A/A	G/G	FAM: AGCAGCGTCGGCAAATTTTCTCT VIC: GCAGCGTCGGCAAATTTTCTCC Com: CCATGTCGCCCACGATCACGTAT
*A009353*	70.63	*AX108860278*	553418567	A/A	G/G	FAM: GCTCATGTCTCTTTCCCTGCAT VIC: CTCATGTCTCTTTCCCTGCAC Com: GCACCAAAGTGTCGCAGGCT
*A009352*	72.57	*AX110476153*	554404315	G/G	C/C	FAM: ATCGGCACGTAAGAGACCTCAC VIC: ATCGGCACGTAAGAGACCTCAG Com: CTCGTGCTGACATGGTTCAGTACA
*A009177*	73.66	*AX111845176*	554025909	C/C	T/T	FAM: ATACCCTCACCAACACCCCG VIC: CATACCCTCACCAACACCCCA Com: GCTGGCTCACTACATTCTTCCACTT
*A009711*	88.1	*AX111543252*	566738033	G/G	T/T	FAM: TGGTGATTAGCATCATCGGAATGG VIC: TTGGTGATTAGCATCATCGGAATGT Com: ATCAAATCTATCGAGTTAAAGCTGCCCAA
*A009180*	105.54	*AX111453429*	573611727	T/T	G/G	FAM: TAAGTGAATTTTTAAAGTTCGCATACCCT VIC: AGTGAATTTTTAAAGTTCGCATACCCC Com: CAACGGCGTACCCCGGATTTTAAAT
*A009715*	123.4	*AX109868172*	584987956	G/G	A/A	FAM: ACTGACTCTAGCTTGATGACACG VIC: CACTGACTCTAGCTTGATGACACT Com: ATGGCCCCCACGAGTCAAAAACATT
*A009725*	147.71	*AX94407298*	597069714	G/G	T/T	FAM: ACAAGTATTCAGCCTCTTTGCCAC VIC: CACAAGTATTCAGCCTCTTTGCCAA Com: GCAATATGGAAGCCTACACTCCCTT
*A009716*	149.21	*AX111561378*	596719275	T/T	G/G	FAM: GTTGCTATGTAACGGAATAAGAACG VIC: CGTTGCTATGTAACGGAATAAGAACT Com: CCAAATAGAAGTATCACTTGAACAATGCTT

**Table 4:** Primers used for the qRT-PCR and cDNA cloning.

Locus	Primer Sequences (5′–3′)	Predicted Product Size to Reference (bp)
*β-actin*	Forward: AGTGGACGCACAACAGGTA Reverse: GTCAAGACGAAGGATGGCA	105
*TraesCS3D01G466100*	Forward: CACCAAATGCTTCCACCTA Reverse: GGCACCAACGACCTACTAC	173
*TraesCS3D01G466100*	Forward: ACACGGGAAAGTAGCT Reverse: GAGACAAGCACGGAGA	2535

## Data Availability

The data presented in this study are available on request from the corresponding author.
